# Control of Radiative Exciton Recombination by Charge Transfer Induced Surface Dipoles in MoS_2_ and WS_2_ Monolayers

**DOI:** 10.1038/srep24105

**Published:** 2016-04-07

**Authors:** Peng Hu, Jun Ye, Xuexia He, Kezhao Du, Keke K. Zhang, Xingzhi Wang, Qihua Xiong, Zheng Liu, Hui Jiang, Christian Kloc

**Affiliations:** 1 School of Materials Science and Engineering, Nanyang Technological University, 639798 Singapore; 2 Institute of High Performance Computing, Agency for Science, Technology and Research, 138632 Singapore; 3 School of Physical and Mathematical Sciences, Nanyang Technological University, 637371 Singapore

## Abstract

Due to the two dimensional confinement of electrons in a monolayer of 2D materials, the properties of monolayer can be controlled by electrical field formed on the monolayer surface. F_4_TCNQ was evaporated on MoS_2_ and WS_2_ monolayer forming dipoles between strong acceptor, F_4_TCNQ, and monolayers of MoS_2_ or WS_2_. The strong acceptor attracts electrons (charge transfer) and decreases the number of the ionized excitons. Free excitons undergo radiative recombination in both MoS_2_ and WS_2_. Moreover, the photoluminescence enhancement is stronger in WS_2_ where the exciton-phonon coupling is weaker. The theoretical model indicates that the surface dipole controls the radiative exciton recombination and enhances photoluminescence radiation. Deposition of F_4_TCNQ on the 2D monolayers enables a convenient control of the radiative exciton recombination and leads to the applications of these materials in lasers or LEDs.

When an exciton, a quasiparticle consisting of an electron and a hole bound together by simple Coulomb interaction, recombines, i) photoluminescence occurs in the case of weak exciton-phonon coupling or ii) exciton recombines radiation-less increasing the phonon energy if this coupling is strong. In a monolayer of transition metal dichalcogenides (TMD), or a two dimensional electron gas, excitons can interact with free electrons forming charged excitons also known as trions, quasiparticles composed of two electrons and a hole^1^. Due to the presence of these tightly bound negative trions, the number of non-bounded excitons available for radiative recombination is limited and the photoluminescence is suppressed. In other words, the non-bounded excitons cannot radiative recombine producing photon (light) because they are bonded with free electrons forming trions^2^,^3^,^4^,^5^,^6^. Therefore, to increase the photoluminescence, the concentration of trions needs to be reduced. A strong electrical field formed by gate electrode on the two-dimensional (2D) layer of TMD or dipoles on the surface of TMD monolayer can reduce the trion concentration^1^.

In previous studies, a chemical doping method was used to enhance the photoluminescence by interaction of TMD monolayer with acceptor in solvent^2^,^5^. Furthermore, some reports studied PL and optical properties controlled by the charge transfer between MoS_2_ and metal nanoparticle^7^ or graphene quantum dots^8^. It was shown that not only the PL intensity has been changed, but also the phase transition in MoS_2_ monolayer is caused by charge transfer^9^. In this work, we evaporate 2,3,5,6-tetrafluoro-7,7,8,8-tetracyanoquinodimethane (F_4_TCNQ) on TMD monolayer forming dipoles between strong acceptor, F_4_TCNQ and monolayers of MoS_2_ or WS_2_. The strong acceptor attracts electrons (charge transfer) and decreases the number of the ionized excitons. Free excitons undergo radiative recombination in both MoS_2_ and WS_2_. Moreover, the photoluminescence enhancement is stronger in WS_2_ where the exciton-phonon coupling is weaker. No solvent was used, which provide a clean system to compare to theoretical calculations. The theoretical model indicates that the surface dipole is controlling the radiative exciton recombination, which further increases the photoluminescence.

## Results

### MoS_2_ and WS_2_ monolayer growth and characterization

**Figure 1:**
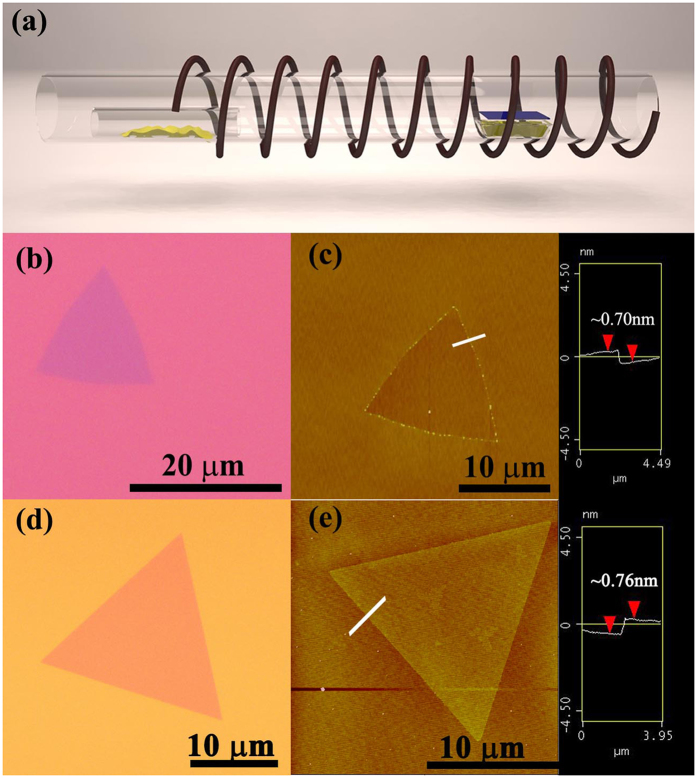
(**a**) Monolayer WS_2_ and MoS_2_ growth apparatus. (**b**) Optical image of triangle monolayer WS_2_. (**c**) AFM image of a monolayer WS_2_ on a SiO_2_/Si substrate and the corresponding section analysis. (**d**) Optical image of triangle monolayer MoS_2_. (**e**) AFM image of a monolayer MoS_2_ on a SiO_2_/Si substrate and the corresponding section analysis.

A triangle monolayer of WS_2_ and MoS_2_ was grown with the chemical vapor deposition (CVD) method onto a SiO_2_/Si wafer. The monolayer growth apparatus is shown in Fig. 1(a). Triangular WS_2_ and MoS_2_ were grown at random locations on the substrate. Figure 1(b,d) show the optical images of the WS_2_ and MoS_2_, respectively. The thickness of the WS_2_ and MoS_2_ was determined by atomic force microscopy (AFM), as shown in Fig. 1(c,e). The AFM images indicate that both the WS_2_ and MoS_2_ have a smooth surface. The cross section height of the WS_2_ and MoS_2_ is approximately 0.70 nm and 0.76 nm, respectively, which corresponds to the monolayers of WS_2_ 5,10 and MoS_2_ 11,12,13,14.

**Figure 2:**
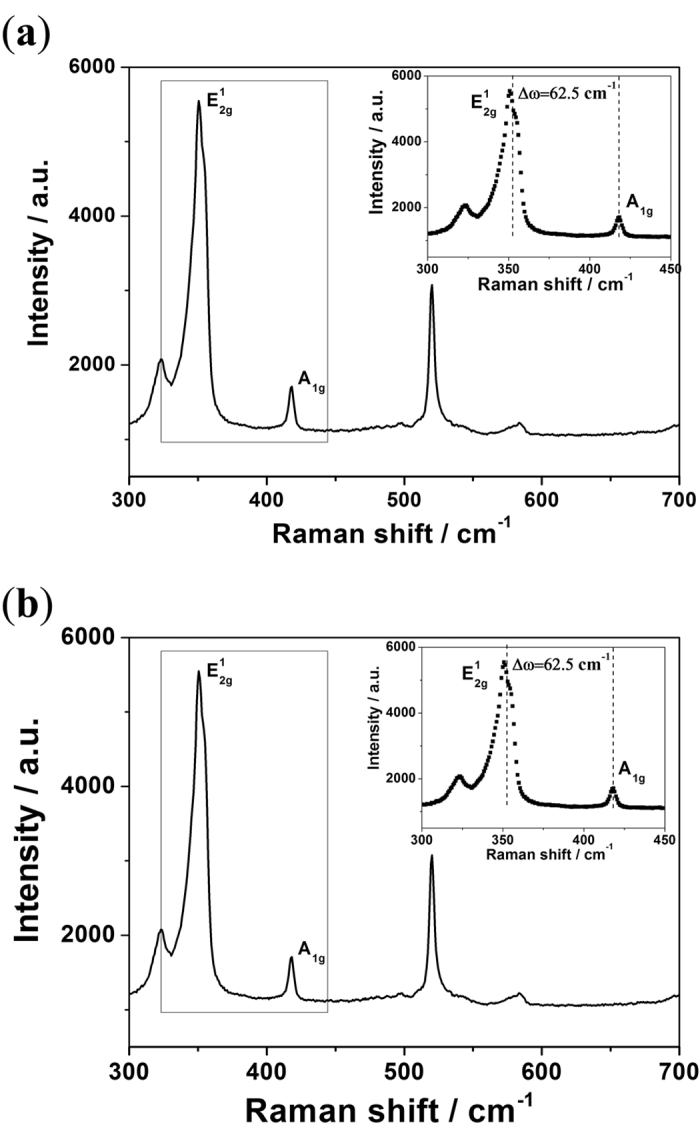
Raman spectra of a CVD-grown WS_2_ monolayer (**a**) and MoS_2_ monolayer (**b**). The inset shows the energy difference between the Raman E_2g_^1^ and A_1g_ modes.

The monolayer structure of WS_2_ and MoS_2_ is further confirmed by the Raman spectrum shown in Fig. 2. The E_2g_^1^ and A_1g_ modes of monolayer WS_2_ are located at approximately 355 and 417 cm^−1^, respectively^15^,^16^,^17^,^18^. With the number of layers increased, the in-plane vibrational E_2g_^1^ is slightly red-shifted, and the out-of-plane A_1g_ mode is blue-shifted. The energy difference between the Raman E_2g_^1^ and A_1g_ modes increased with the layer number. Thus, the energy difference can be used to identify the number of layers of WS_2_. The energy difference shown in Fig. 2(a) is 62.5 cm^−1^, which coincides with previous reports for monolayer WS_2_ 15,19. The same phenomenon is also observed in monolayer MoS_2_. The in-plane vibrational E_2g_^1^ phonon mode is ∼385 cm^−1^, and the out-of-plane A_1g_ mode is ∼404 cm^−1^. The energy difference between the two modes is also dependent on the number of layers of MoS_2_. The energy difference between the two modes is 18.2 cm^−1^, as shown in Fig. 2(b), indicating that the MoS_2_ is a monolayer^2^,^14^,^17^,^20^,^21^.

### Photoluminescence intensity after F_4_TCNQ was deposited onto monolayer MoS_2_/WS_2_

**Figure 3:**
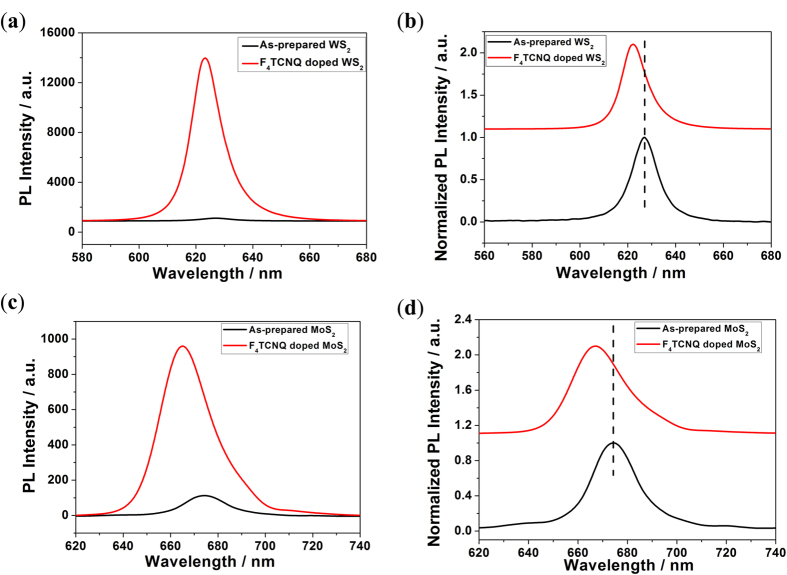
PL spectra of monolayer WS_2_ (**a**) and monolayer MoS_2_ (**c**) before and after F_4_TCNQ doping. PL peak shift of monolayer WS_2_ (**b**) and monolayer MoS_2_ (**d**) before and after F_4_TCNQ doping.

Figure 3(a) shows that the PL intensity before and after F_4_TCNQ was deposited onto monolayer WS_2_. The PL intensity is approximately fifty times higher after the F_4_TCNQ deposition. The position of the PL peak of monolayer WS_2_ is slightly blue-shifted, while the peak shape did not change, as shown in Fig. 3(b). The PL intensity is also increased by approximately ten times after the F_4_TCNQ is deposited on MoS_2_ monolayer. The positon of the peak is also slightly blue-shifted, but the shape is not changed, as is shown in Fig. 3(c,d).

## Discussion

**Figure 4:**
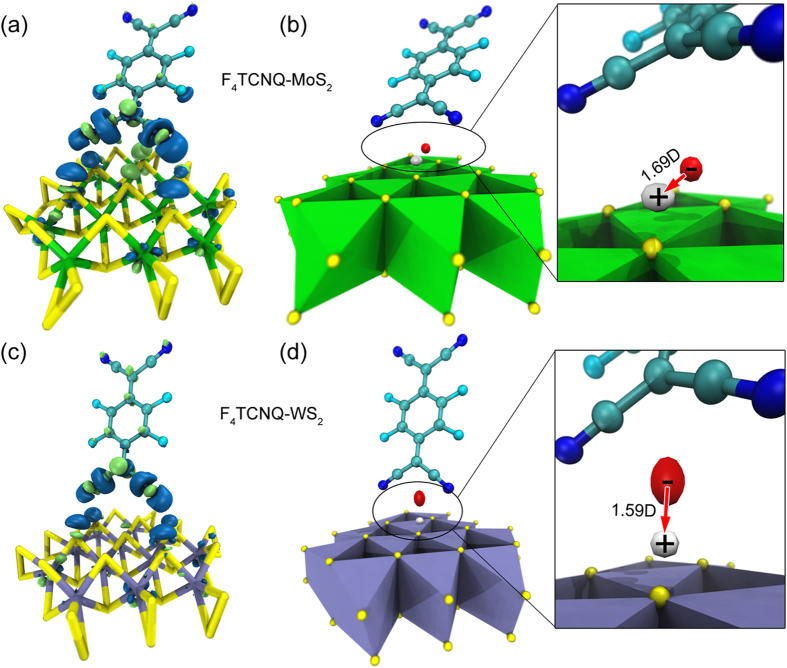
(**a**) Electron density differences (with ± isovalues of 0.005 a.u.) and (**b**) barycenters (with ± isovalues of 0.0001 a.u.) of an F_4_TCNQ-doped MoS_2_ cluster model. Electron density differences (with ± isovalues of 0.005 a.u.) and barycenters (with ± isovalues of 0.0001 a.u.) for F_4_TCNQ-doped WS_2_ cluster model are given in (**c**,**d**), respectively. Green and blue isosurfaces indicate positive and negative values in electron density differences, while red and white isosurfaces indicate plus (electron density increase) and minus (electron density depletion) values of barycenters in (**b**,**d**). The dipole moment variation before and after charge transfer are also displayed in the enlarged view of (**c**,**d**), with ± sign indicating virtual charge of the barycenters due to electron density depletion/increase.

To understand the charge transfer from MoS_2_/WS_2_ to F_4_TCNQ, we performed density functional (DFT) calculations^22^ on the model systems shown in Fig. 4. In both the F_4_TCNQ-doped MoS_2_ and WS_2_ cases, electron density depletion (as indicated by the white isosurfaces) were found in the interface regions where the nitrogen atoms in the F_4_TCNQ molecules are closest to the surface sulfur atoms in MoS_2_ and WS_2_, as shown in Fig. 4(a,c), respectively. The electron density depletion in the MoS_2_ layer is slightly greater than that in the WS_2_ layer according to the electron density difference plots. An electron density increase (red isosurfaces) is observed on the F_4_TCNQ molecules in both cases, as shown in Fig. 4(a,c). Charge transfer occurs around the interface regions in both cases. The energy level of F_4_TCNQ and MoS_2_/WS_2_ are shown in Supporting Information - Figure S1. In addition, the barycenters of the holes (white isosurfaces) shown in Fig. 4(b,d) clearly suggest that the holes are close to the MoS_2_ and WS_2_ surfaces, indicating the charge transfer from MoS_2_ or WS_2_ to F_4_TCNQ. The charge transfer distance (D_CT_) between MoS_2_ and F_4_TCNQ (calculated D_CT_ = 1.299 Å) is shorter than that between WS_2_ and F_4_TCNQ (calculated D_CT_ = 1.391 Å). However the charge transfer direction, indicated by arrows on the Fig. 4(b,d) is determined by the orientation of F_4_TCNQ molecule relative to the surface of the TMD monolayer.

According to our discussion in introduction, the charge transfer between MoS_2_ or WS_2_ monolayer and acceptor, F_4_TCNQ forms dipole layers at interface and reduces the ratio of charged exciton to neutral excitons. Therefore, the photoluminescence (PL) of both materials was enhanced due to the charge transfer.

The experimental results for PL enhancement for both MoS_2_ and WS_2_ are similar to the earlier reported photoluminescence of MoS_2_ and WS_2_ doped with F_4_TCNQ from solution^2^,^5^. In previous studies^2^,^5^, mechanical exfoliated MoS_2_ and WS_2_ were used. Mechanical exfoliation is the easiest and the fastest method to obtain monolayers of MoS_2_ and WS_2_. However, only a small portion of MoS_2_ and WS_2_ crystals are exfoliate to monolayers, leaving a majority of samples as thicker flakes. In this study, we used the chemical vapor deposition to obtain large-area, high-quality monolayers of MoS_2_ and WS_2_. Therefore, after the F_4_TCNQ deposition, our photoluminescence intensity of WS_2_ and MoS_2_ is approximately fifty times and ten times higher, respectively. Compared to the solution-based chemical doping on MoS_2_ monolayer^2^, the PL increases approximately three times. During the vacuum deposition of F_4_TCNQ on MoS_2_ monolayer, there is no solvent contamination and interaction between MoS_2_ and F_4_TCNQ and therefore the PL increases stronger.

**Figure 5:**
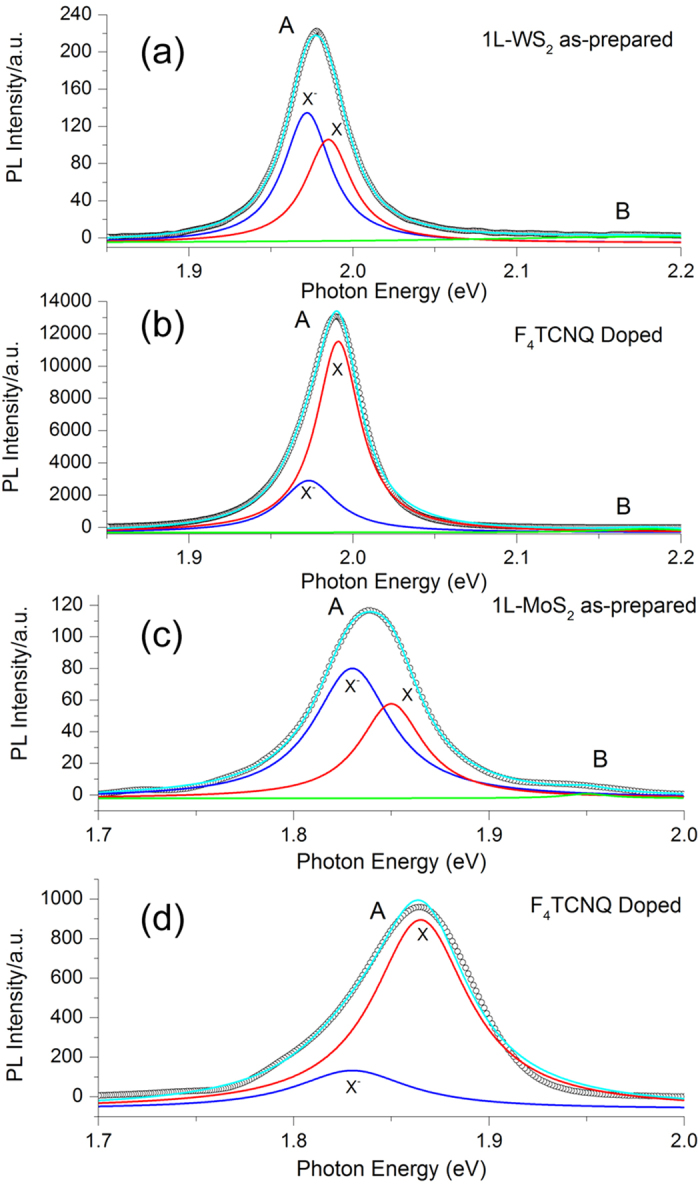
Fitted PL spectra of monolayer WS_2_ (**a**) before and (**b**) after F_4_TCNQ doping. Fitted PL spectra of monolayer MoS_2_ (**c**) before and after F_4_TCNQ doping (**d**). Lorentzian functions were used to fit the A and B peaks, with A peaks assumed to be composed of trions (X^−^) and excitons (X).

The optical properties of MoS_2_ and WS_2_, especially the photoluminescence, are affected by the number of layers^6^,^23^. Few-layered MoS_2_ and WS_2_ have an indirect band gap and show low photoluminescence, while monolayers of MoS_2_ and WS_2_ have a direct bandgap and strong photoluminescence^23^,^24^. To understand the effects of charge transfer, the photoluminescence peaks, which are due to the direct band gap transition, have been analyzed by fitting them with photoluminescence from trions and photoluminescence from neutral excitons represented by two Lorentzian functions, as shown in Fig. 5. For the all cases studied, the photoluminescence signal can be decomposed as A and B peaks, but the intensity of the B peak is negligible. The A peak can be further decomposed to trion (X^−^) and exciton (X) components. Peak positions from the fitting can be found in Table 1. The exciton binding energy of MoS_2_ and WS_2_ (1.85 eV and 1.985 eV) was determined in our work. The trion spectral weight I_X_^−^/I_total_ was also calculated and listed in Table 1.

For both WS_2_ and MoS_2_, the trion spectral weight I_X_^−^/I_total_ decreases after charge transfer, as shown in Table 1. This indicates that the charge transfer significantly decreases the concentration of trions by transferring electrons from the trions into acceptors, thereby enhancing the photoluminescence.

Upon the deposition of F_4_TCNQ on the monolayers, the charge transfer reaches a maximum because the trion spectral weight reaches the saturation region at approximately 0.2. We observe that the peaks for the corresponding X^−^ and X of PL are sharper for WS_2_ than for MoS_2_. The wider peak width is associated with a stronger coupling strength or a larger Huang-Rhys factor S for a typical semiconductor^25^,^26^, so that we may ascribe the narrower PL peaks for WS_2_ samples compared to MoS_2_ as indicative of slightly weaker exciton-phonon coupling^27^. After charge transfer to the F_4_TCNQ molecules, the peak width change is almost the same. The weaker exciton-phonon scattering of WS_2_ results in narrower PL peaks with a larger amplitude.

The DFT calculated electron transferred from MoS_2_ and WS_2_ to F_4_TCNQ was 0.271 and 0.237, respectively. These data are in good agreement with the trion spectral weight data (Table 1). Larger amount of charge transferred causes more trions to be dissociated to excitons, thereby leading to a lower trion spectral weight. The surface dipole is formed due to the charge transferred from MoS_2_/WS_2_ to F_4_TCNQ. The amount of transferred charge can control the intensity and position of PL. The adding electrons to or withdrawing electrons from the 2D monolayer decreases^7^,^8^,^9^ or increases (this work) the intensity of PL.

It is worth to notice that a dipoles formed by charge transfer to acceptor deposited directly on 2D semiconductor is comparable to dipoles formed by Helmholtz double layer in a electrolyte double layer transistor (EDLT), where gate is a reference electrode in an ionic organic liquid. In an EDLT, the number of induced charges is in the transistor channel is in the range of 10^14^ 1/cm^2^. It is almost one order of magnitude larger than the charge induced by the layer of dipoles in our experiment, but two orders of magnitude larger than the charge induced in MoS_2_ transistor with 280 nm SiO_2_ and gate voltage of −70 V^1^. Such high concentration of charge induced in a monolayer of TMD semiconductors should lead to correlated effects like ferromagnetism or to superconductivity in EDLT MoS_2_ system^28^. Additionally acceptor layer deposited on the surface on 2D semiconductors can be considered as a stable gate that doesn’t required additional connector for gate voltage.

## Conclusion

In summary, triangle monolayer WS_2_ and MoS_2_ were grown using the chemical vapor deposition (CVD) method. The formation of the monolayers was confirmed by both AFM and Raman spectra. The PL increased after a thin layer of F_4_TCNQ was deposited on the surface of the WS_2_ and MoS_2_ monolayers. The ratio of charged excitons, trions, to neutral excitons decreases due to the charge transfer from monolayer WS_2_ and MoS_2_ to strong acceptor, F_4_TCNQ. The weaker exciton-phonon interaction of WS_2_ results in narrower PL peaks with larger amplitudes than in MoS_2_ where this interaction is strong. Acceptors or donators deposited on the surface of MoS_2_ or WS_2_ and also on other 2D monolayers provides an effective mechanism for controlling the electron distribution in such heterojunctions. In this way, it is a convenient method of tuning the optoelectronic properties of 2D materials and leads to the application of these materials in lasers or LEDs.

## Methods

### Chemicals and materials

WO_3_ (>99.5%), MoO_3_ (>99.5%) and sulfur (>99.95%) powders were purchased from Sigma-Aldrich and used without any purification. F_4_TCNQ (>99.5%) was purchased from Jilin OLED Materials Tech. Co. Ltd. and purified at 220 °C via physical vapor transport (PVT).

### Preparation of MoS_2_ and WS_2_ monolayers

For both the triangular shaped MoS_2_ and WS_2_ monolayers, we used the same method of chemical vapor deposition (CVD). The growth process for the two materials is almost the same, with the only difference being the precursor. Commercially available SiO_2_/Si substrates were used in this study. All the substrates were successively cleaned with acetone, methanol, H_2_O_2_/H_2_SO_4_ (1 volume/4 volume) and distilled water in an ultrasonic bath for 5 min and then dried in ambient N_2_. First, fine WO_3_ or MoO_3_ powder was spread on the bottom of the crucible. One piece of SiO_2_/Si substrate (1 × 1 cm) was placed face-down on the crucible, and the crucible was put in the center of the growth furnace. Another small crucible with approximately 50 mg sulfur powder was put in another part of the furnace near the gas input side at a temperature of 200 °C. The furnace was heated to 750 °C at 25 °C/min and then maintained at that temperature for 20 min before naturally being cooled down to room temperature. Argon gas was provided during the whole growth process at 60 sccm.

### Preparation of F_4_TCNQ layers on WS_2_ and MoS_2_ monolayers

2-nm F_4_TCNQ was deposited on the WS_2_ and MoS_2_ monolayers by the evaporation of F_4_TCNQ in a Tectra mini-coater (Germany) with a deposition rate of 0.1 angstrom per second.

### Characterization

Photoluminescence was measured at the same area before and after F_4_TCNQ deposition. Both the laser beams (solid-state laser, 473 nm and Nd:YAG solid-state laser, 532 nm) were collimated and focused through a ×100 objective onto the sample surface. All the spectra were collected using a confocal triple-grating spectrometer (Horiba-JY T64000). Raman spectra were recorded using a Renishaw Raman microscope configured with a charge-coupled device array detector with the excitation laser line of 532 nm. Atomic force microscopy was performed on a Digital Instruments 3100.

### Density functional theory calculations

The geometry of the F_4_TCNQ on the surface of the MoS_2_/WS_2_ was optimized using the DMol^3^ 29,30 with the dispersion-corrected (OBS) PW91 (GGA) functional at the level of the DNP basis set. The geometry of the models is regarded as converged when the total energy difference is less than 1 × 10^−5 ^Ha, the total force difference is less than 4 × 10^−3^ Ha/Å, and the maximum displacement of atoms is less than 5 × 10^−3^ Å during the optimization. The optimized geometries of the models were subsequently fed into the ORCA 3.0.3 package^31^ to perform single-point energy calculations (with SCF convergence criteria set as 1 × 10^−6 ^Ha) at the level of B3LYP/6-31G(d,p) (with Mo and W atoms treated using SDD effective core potentials^32^). To facilitate the charge transfer analysis, the MultiWFN 3.3.7 package^33^ was used to calculate the charge transfer based on electron density difference.

## Additional Information

**How to cite this article**: Hu, P. *et al*. Control of Radiative Exciton Recombination by Charge Transfer Induced Surface Dipoles in MoS_2_ and WS_2_ Monolayers. *Sci. Rep.* **6**, 24105; doi: 10.1038/srep24105 (2016).

## Supplementary Material

Supplementary Information

## Figures and Tables

**Table 1:** Peak position and width for Lorentzian functions used to fit PL peak A in Fig. 5.

Sample	Peak name	Peak Position (eV)	FWHM(meV)	I_X−_/I_total_
1 L WS_2_ as-prepared	X^−^ trion	1.972 (1.96)	35	0.63
	X exciton	1.985	35	
F_4_TCNQ doped 1 L WS_2_	X^−^ trion	1.973 (1.98)	42	0.25
	X exciton	1.991 (2.02)	33	
1 L MoS_2_ as-prepared	X^−^ trion	1.83 (1.84)	48	0.70
	X exciton	1.85 (1.88)	40	
F_4_TCNQ doped 1 L MoS_2_	X^−^ trion	1.83 (1.84)	70	0.20
	X exciton	1.865 (1.88)	60	

(The values in brackets for peak positions were previously reported^2^,^5^).
