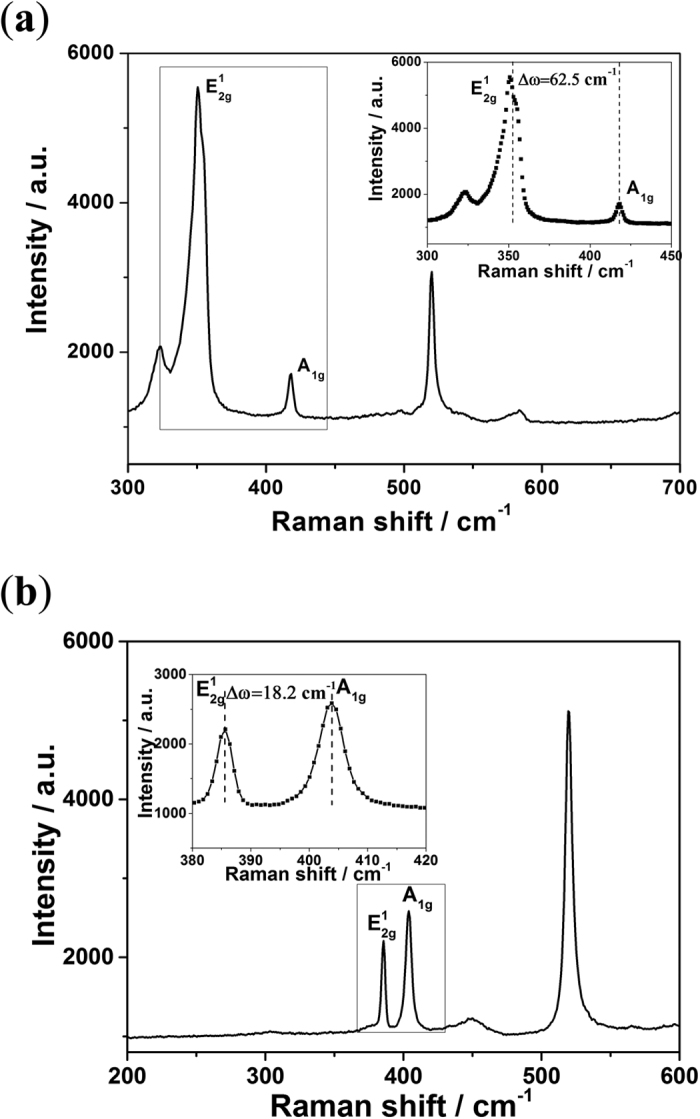# Corrigendum: Control of Radiative Exciton Recombination by Charge Transfer Induced Surface Dipoles in MoS_2_ and WS_2_ Monolayers

**DOI:** 10.1038/srep46951

**Published:** 2018-04-05

**Authors:** Peng Hu, Jun Ye, Xuexia He, Kezhao Du, Keke K. Zhang, Xingzhi Wang, Qihua Xiong, Zheng Liu, Hui Jiang, Christian Kloc

Scientific Reports 6: Article number: 24105; 10.1038/srep24105 published online: 04 07 2016; updated: 04 05 2018.

This article contains an error in Figure 2, where the same image was inadvertently shown in both panel (a) and (b). The correct Figure 2 appears below:

## Figures and Tables

**Figure 2:**