# Supplementary material for: Control of Radiative Exciton Recombination by Charge Transfer Induced Surface Dipoles in MoS2 and WS2 Monolayers
Source: Sci Rep. 2016 Apr 7;6:24105. doi: 10.1038/srep24105 (PMC4823649; doi:10.1038/srep24105)
Supplement: Supplementary Information [file srep24105-s1.doc]

**Supporting Information**

Control of Radiative Exciton Recombination by Charge Transfer Induced Surface Dipoles in MoS_2_ and WS_2_ Monolayers

Peng Hu,^1^ Jun Ye,^2^ Xuexia He,^1^ Kezhao Du,^1^ Keke K. Zhang,^1^ Xingzhi Wang,^3^ Qihua Xiong,^3^ Zheng Liu,^1,*^ Hui Jiang,^1,*^ and Christian Kloc ^1,*^

1. School of Materials Science and Engineering, Nanyang Technological University, 639798 Singapore.

2. Institute of High Performance Computing, Agency for Science, Technology and Research, 138632 Singapore Address here.

3. School of Physical and Mathematical Sciences, Nanyang Technological University, 637371 Singapore

Figure S1. The energy level diagram of (a) F_4_TCNQ and MoS_2_; (b) F_4_TCNQ and WS_2_.

**
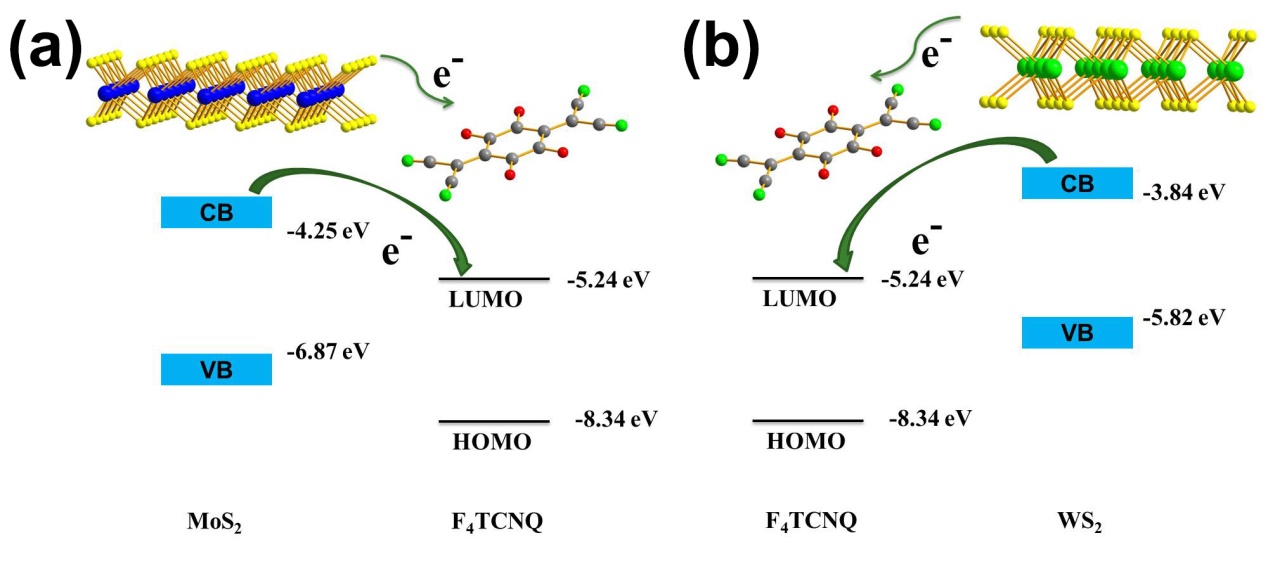
**
